# Factors contributing to innovation readiness in health care organizations: a scoping review

**DOI:** 10.1186/s12913-022-08185-x

**Published:** 2022-08-05

**Authors:** Monique W. van den Hoed, Ramona Backhaus, Erica de Vries, Jan P. H. Hamers, Ramon Daniëls

**Affiliations:** 1 Department of Health Services Research, Care and Public Health Research Institute, Maastricht University, Maastricht, The Netherlands; 2 Living Lab in Ageing and Long-Term Care, Maastricht, The Netherlands; 3 Expertise Centre for Innovative Care and Technology, Research Centre for Assistive Technology in Care, Zuyd University of Applied Sciences, Heerlen, The Netherlands

**Keywords:** Innovation, Innovation readiness, Organizational readiness, Scoping review, Health care, Innovative climate

## Abstract

**Background:**

Increasing innovation readiness of healthcare organizations is necessary to meet upcoming challenges, including population aging, staff shortages and reduced funding. Health care organizations differ in the extent to which they are innovation ready. This review aims to clarify the concept of innovation readiness and identify which factors contribute to innovation readiness in health care organizations.

**Methods:**

A scoping review was conducted based on the framework from Arksey and O’Malley. PubMed/MEDLINE, CINAHL and Web of Science were searched for studies that (a) aimed to contribute to scientific knowledge about innovation readiness of health care organizations, (b) were peer-reviewed, (c) reported empirical data and (d) were written in English, Dutch or German. Factors researched in the included studies were bundled into 4 overarching main factors and 10 sub-factors.

**Results:**

Of the 6,208 studies identified, 44 were included. The majority (*n* = 36) of the studies had been conducted since 2011 and almost half of the studies (*n* = 19) were performed in hospitals. Of the 44 studies, 21 researched factors contributing to innovation readiness in the implementation stage of the innovation process. The authors used a variety of words and descriptions addressing innovation readiness, with hardly any theoretical frameworks for innovation readiness presented. Four main factors and 10 sub-factors contributing to the innovation readiness of health care organizations were summarized: strategic course for innovation, climate for innovation, leadership for innovation and commitment to innovation. Climate for innovation (*n *= 16) was studied the most and individual commitment to innovation (*n* = 6) was the least studied.

**Conclusion:**

Our study identified four main factors contributing to the innovation readiness of health care organizations. Research into innovation readiness of health care organizations is a rather new field. Future research could be directed towards defining the concept of innovation readiness and the development of a framework for innovation readiness. More understanding of the interplay of factors contributing to innovation readiness in all stages of the innovation process and in diverse health care settings can support health care managers to structurally embed innovation. This review contributes to the first stage of theory building on factors contributing to innovation readiness of health care organizations.

**Supplementary Information:**

The online version contains supplementary material available at 10.1186/s12913-022-08185-x.

## Background

Upcoming challenges such as population aging, staff shortages and reduced funding compel health care organizations to innovate [1–5]. Greenhalgh et al. [6] see innovation as “a novel set of behaviors, routines and ways of working that are discontinuous with previous practice, are directed at improving health outcomes, administrative efficiency, cost effectiveness, or user experience and that are implemented by planned and coordinated actions.” Success in innovating depends on an interplay of factors [2, 7] and needs to be planned [8, 9]. Health care organizations show large differences in the extent to which they are capable of innovating [10–13]. Increasing the readiness of health care organizations for innovation is required to meet the challenges they face [1–5, 13].

Although of great importance, embedding innovation structurally in health care organizations is not simple [14–16]. Organizations encounter difficulties in structuring their innovation processes [3, 17, 18], while existing procedures and regulations often restrain innovative initiatives [10, 19]; moving to a state of readiness for innovation differs from preparing to introduce a specific innovation [2]. While many studies focus on the implementation of specific treatment or e-health innovations [20–24], scientific knowledge about what is needed for health care organizations to become innovation ready is limited [2, 25, 26]. Scientists have only recently focused attention on organizational readiness for innovation in health care settings [4].

Health care organizations can greatly benefit from knowledge about how to prepare to succeed in any type of innovation to meet strategic challenges [10, 27–29]. To the best of our knowledge, there are no overviews that specifically address factors contributing to innovation readiness in health care organizations. Therefore, we conducted a scoping review (1) to clarify the concept of innovation readiness and (2) to identify available research on the factors contributing to innovation readiness in health care organizations. Innovation readiness in business and health care received various explanations, with no generally accepted explanation in the literature [2, 4, 30]. Innovation readiness is explicitly referred to as the ability to innovate by Zerfass et al. [31].

In our view innovation readiness indicates the level of maturity of an organization to succeed in any type of innovation. Moving to a state of readiness for innovation has a broader scope than the introduction of a specific innovation [32]. Innovation readiness comprehends the entire innovation cycle while organizational readiness for change specifically measures the successful adoption of new innovations [15].

## Methods

The scoping review was conducted based on the framework from Arksey and O’Malley [33] and the Joanna Briggs Institute’s recommendations for conducting scoping reviews [34]. The reporting is according to the Preferred Reporting Items for Systematic Reviews and Meta-Analyses extension for Scoping Reviews (PRISMA-ScR) checklist; see Additional File 1 for PRISMA Checklist [35].

### Identifying the research question

The scoping review question guided the development of the inclusion criteria and provided a clear structure for the development of the scoping review. As with the title, the question incorporates the population, concept, and context elements [36]: What is known in scientific literature about: (1) the concept of innovation readiness (definitions, theories, frameworks) (2) the factors that are studied contributing to innovation readiness in health care organizations. The review aims to map a range of factors that contribute to innovation readiness (concept) for health care organizations (population and context). In this review the population and the context (health care organizations) overlap. For this study in the health care context, we consider ‘innovation readiness’ to be the level of maturity of an organization to succeed in any type of innovation and ‘becoming or being innovation ready’ as an ongoing, coherent and tuned process consisting of planned and coordinated actions to optimize the entire innovation cycle.

### Identifying relevant studies

The literature search was executed on June 11, 2021, within three databases: PubMed/MEDLINE, (biomedical literature from MEDLINE, life science journals, and online books), CINAHL (focus on nursing journals) and Web of Science (scholarly journals in science, social sciences, and humanities disciplines) (see Additional File 2: Detailed search terms used in the PubMed search). Web of Science was added as a database to identify studies about innovation readiness published in economic and innovation journals.

In March 2021 the term “innovation readiness” was explored via a preliminary search in Google scholar and PubMed/MEDLINE to identify relevant and related concepts and terms. Innovation readiness (the term itself and the broader meaning) appeared in a diversity of related terms e.g., innovation/organization capacity/potential, ready for and in a diversity of scientific journals (e.g. medicine, health, innovation, management, economy).

The search strategy is based on the main concept term: innovation readiness for PubMed/Medline and CINAHL and on the main concept terms: innovation readiness and health care for Web of Science. Innovation readiness is a nonspecific search term in all three databases. We studied recent systematic and scoping reviews for the search strategies employed for operationalizing the innovation readiness concept. To formulate the search strings, relevant keywords and synonyms were identified for each concept term in addition to the controlled vocabulary terms (such as the MeSH headings in PubMed).

Reference lists of articles that fulfilled the inclusion criteria were searched to identify additional papers. The final search strategy is available as appendix to the review. As the database Web of Science includes articles of a diverse setting (broader than the health care setting of Pubmed and Cinahl) we operationalized the health care concept via studying recent health care research, which we adapted for our use.

The search for this scoping review was quite iterative as in time we became more familiar with the additional keywords and sources, and potentially useful search terms discovered and incorporated into the search strategy. The search strategy was discussed on several occasions by the research team (also authors) as well as reviewed twice by a research librarian from Maastricht University.

### Eligibility criteria and study selection

Studies were included if the study (a) aimed to contribute to scientific knowledge about innovation readiness of health care organizations, (b) was peer-reviewed, (c) reported empirical data and (d) was written in English, Dutch or German. We included German and Dutch, besides English (authors are fluent in these languages) to broaden the scope and the number of available articles. The results from the database search were merged and duplicates were removed using reference-management software (Endnote).

**Fig. 1:**
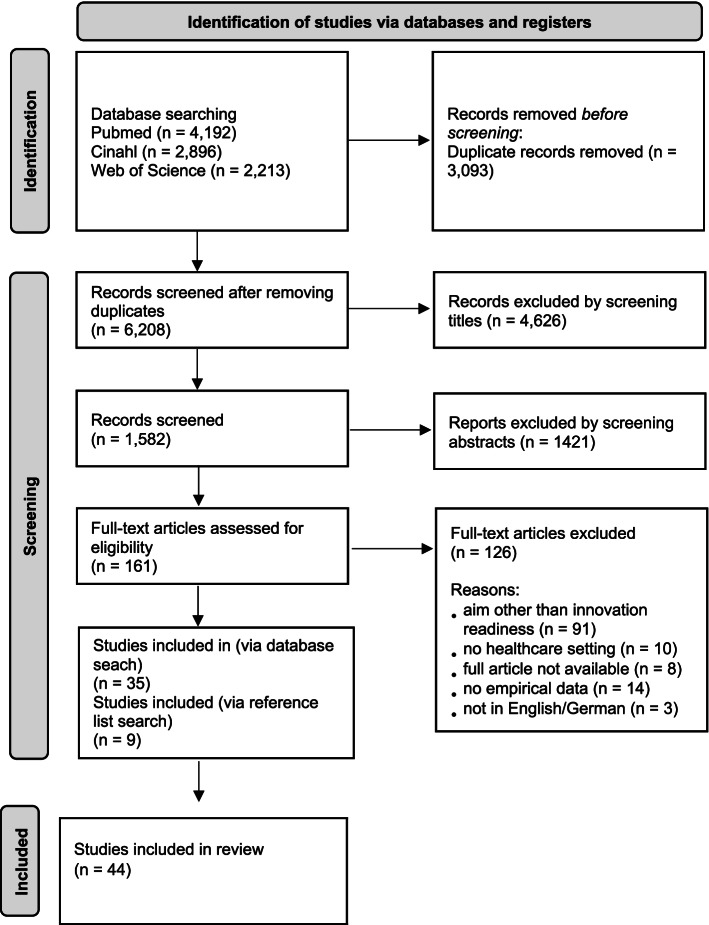
PRISMA 2020 flow diagram of search of databases, identification and inclusion for the review

Author MH screened all articles by titles and by abstracts for eligibility and author RB screened a random selection of 10%. Both authors compared their assessment decisions, logged in review software Rayyan and resolved discrepancies through consensus. These phases produced 161 ‘included studies’. In the next phase (screening via full text), two authors (MH, EV) independently screened and discussed 100% of the full text of the retrieved studies and scored them as ‘include’, 'possibly include' or 'exclude'. Reference lists of included studies were searched by the author MH to identity additional studies. All extracted data were discussed within the research team. The selection process followed the PRSIMA 202 flow chart (see Fig. 1).

### Data extraction

A data extraction table was developed. Two authors (MH, EV) extracted the data of half of the studies and verified each other’s data. The data charting table consists of author(s), year, country(ies), study aim, design, setting and factor(s). A factor is the key construct of innovation readiness addressed in the study.

### Summarizing and reporting the results

For the first part of the research question, definitions and descriptions of innovation readiness as well as frameworks were extracted from the included studies. A framework (including theoretical models or frameworks related to innovation readiness) demonstrates the understanding of the factors, variables, or constructs and the presumed relationships between them, addressed in the study, often being displayed graphically [37]. For the second part of the research question, two authors (MH, EV) listed the main factors contributing to innovation readiness, as well as the main results of the studies to identify key characteristics of the factors contributing to innovation readiness. Based on the descriptions of factors studied factors were then bundled into 4 overarching main factors and 10 subfactors. Although some sub-factors may relate to more than one main factor, it was decided to assign each sub-factor to one main factor.

## Results

### Characteristics of the studies

The initial search strategy identified 6,208 references after duplicates were removed. After screening using the inclusion criteria, we retained 44 studies: i.e., [20, 38–80]. All articles are written in English. The details of the studies included in the review are presented in Table 1. Studies were published from 1997 onwards, with the majority (almost 90%) being published since 2011. Ten of the included studies were published in either 2020 or 2021. Most studies were conducted in Europe (*n* = 22, 50%) and the USA (*n* = 15, 34%) and in hospitals (*n *= 19, 43%) and long-term care organizations (nursing homes, care providers for the mentally or physically disabled) (*n* = 9, 20%). The research methods show a wide variety, such as group and individual (semi-structured) interviews, the Delphi study, observations of participant and project meetings, focus groups, (grey) literature reviews, prototyping, workshops, focus-groups, case studies, (online) questionnaires and statistical analysis. Qualitative research design was used in 18 studies, quantitative research design in 16 studies and a mixed method design in 10 studies.

**Table 1:** Characteristics of the 44 included studies

Author(s) (year of publication)	Country(ies)	Aim of data collection	Design	Setting	Factor(s) studied (described by authors(s))	Sub-factors
Anvik et al. (2020) [38]	Norway	to investigate the conditions under which learning and innovation occur within nursing homes	qualitative	nursing home	work place learning and innovation	room for learning
Atkinson and Singer (2020) [39]	USA	to examine how interdisciplinary teams developing healthcare innovations manage challenges that may arise from within their organizational context over the lifecycle of the innovation process	qualitative	hospital	constraints in the innovation process	innovation process
Barnett et al. (2011) [40]	UK	to understand how innovators from healthcare organizations made sense of factors that either facilitated or obstructed the innovation implementation and diffusion	qualitative	health care organizations	factors influencing the innovation implementation and diffusion	innovation process
Benson (2019) [41]	UK	develop a set of short generic user-reported related measures to help understand how and why healthcare innovations spread	qualitative	hospital	article included for definition	
Birken et al. (2015) [42]	USA	to assess the influence of top managers’ support on middle managers’ commitment to innovation implementation	mixed-method	health centers	middle managers' commitment to innovation	middle manager’s role
Bunn et al. (2020) [43]	UK	to study researcher’s awareness of organizational context in the collaboration of health care organizations and university researchers on in-house innovation	qualitative	nursing home	organizational context	inter-organizational links
Carpenter et al. (2018) [44]	USA	to study how learning communities can support innovation adoption	qualitative	hospital	learning communities	room for learning
Chuang et al. (2011) [45]	USA	to understand the organizational and relational factors that influence middle managers’ support for the innovation implementation process	qualitative	health care organizations	middle manager support for the innovation process	middle manager’s role
Cramm et al. (2013) [46]	The Netherlands	to explore the importance of a professional’s perceived effectiveness, organizational support, and management support for innovative culture	mixed-method	long-term care	innovative culture	innovative culture
Dohan et al. (2017) [47]	Canada	to investigate the link between technology-related knowledge and skills of healthcare professionals and the ability of healthcare organizations to innovate	mixed-method	paramedics	technology-related skillsets of healthcare professionals	innovative competencies
Emiralioglu and Sonmez (2021) [48]	Turkey	to determine the relation between employees' work environment and innovation support and innovative behaviors of employees	mixed-method	hospital	employees' innovative behavior	innovative behavior
Engle et al. (2017) [49]	USA	to expand the understanding of middle managers’ influence in organizations by looking at their activities	qualitative	veteran medical center	middle managers influence	middle manager’s role
Fleuren et al. (2004) [50]	The Netherlands	to explore which determinants of innovation strategies are reported in the literature	mixed method	implementation experts	(determinants of the) innovation strategy	innovation strategy
García-Goñi et al. (2007) [51]	Spain	to study the differences in behaviour of managers and front-line employees towards innovation and the involvement in the innovation processes	quantitative	public health	role of the middle manager	middle manager’s role
Glover et al. (2020) [52]	Israël	to examine the relationship between unit complexity, autonomy, performance orientation and innovation	mixed-method	hospital	relation between unit complexity and innovation programs	innovation process
Goes and Park (1997) [53]	USA	to conceptualize various types of interorganizational links as opportunities for learning and resource sharing in the pursuit of innovation	mixed-method	hospital	Interorganizational links	inter-organizational links
Gunzel-Jensen et al. (2018) [54]	Denmark	to study the interaction between different types of leadership (transformational, transactional and empowering leadership) and the innovative behavior employees	mixed-method	hospital	leadership (transformational, transactional and empowering)	leadership style
Helfrich et al. (2007) [55]	USA	to study and adapt an organizational framework of innovation implementation (out of manufacturing) for implementation in the health sector	qualitative	clinical research network	organizational framework of innovation implementation effectiveness	innovative culture
Hunter et al. (2021) [56]	USA	to report on a competition-based program designed to accelerate early-stage, innovative health care ideas from within the healthcare organization	qualitative	hospital	innovation program	innovation programm
Hyrkas et al. (2020) [57]	Finland	to develop and test a co-creation model for collaborative innovation, in which companies and healthcare professionals co-create future health care services	qualitative	hospital	colloborative / co-creation innovation process	innovation process
Jacobs et al. (2015) [20]	USA	to examine the innovation implementation framework: the organizational factors that determine effective innovation implementation	quantitative	hospital	innovation implementation (climate)	innovative culture
Jaskyte and Dressler (2005) [58]	USA	to improve innovation models by incorporating organizational culture into the conceptual model along with transformational leadership and size variables	mixed-method	assocation of retarded citizens	organizational culture	innovative culture
Jonsson et al. (2020) [59]	Denmark	to develop and validate a measurement for the relation between distributed leadership agency and innovation by employees	quantitative	hospital	employee-driven innovation	innovative behavior
Joseph (2015) [60]	USA	to explore the experiences of nurse leaders and nurses in a hospital whose patient care mission was stated as innovation	qualitative	hospital	organizational culture and climate	innovative culture
Keown et al. (2014) [61]	UK	to test a framework highlighting the cultural dynamics to support innovation and its diffusion	mixed-method	health care professionals	factors and behaviors that foster innovation	innovative culture
Leal-Rodriguez et al. (2013) [62]	Spain	to test the relationship between knowledge management and the effectiveness of the innovation process	quantitative	hospital	the innovation process	innovation process
Lombardi et al. (2018) [63]	USA	to test a framework to characterize the essential organizational capabilities required for high-impact health care innovation	mixed-method	academic health center	organizational capabilities for high-impact health care innovation	innovation strategy
Masood and Afsar (2017) [64]	Pakistan	examining the influence of the role of leadership to develop and foster innovation	quantitative	hospital	transformational leadership	leadership style
Nieboer and Strating (2012) [65]	The Netherlands	to identify organizational characteristics that explain innovative culture in the (long-term) care sector	quantitative	long-term care	innovative culture	innovative culture
Reed et al. (2012) [66]	USA	to understand the process of patient-centered care innovation undertaken by innovative health care organizations	qualitative	health care professionals	innovation process	innovation process
Renkema et al. (2021) [67]	The Netherlands	to investigate the relationship between high-involvement human resource management, autonomy, affective organizational commitment and innovative behaviors	quantitative	nursing home	innovative behaviour	innovative behavior
Rokstad et al. (2015) [68]	Norway	to investigate the role of leadership in the implementation of innovations	qualitative	nursing home	leadership	leadership style
Saidi et al. (2017) [69]	South Africa	to study the role of space in facilitating innovation	qualitative	hospital	workplace design	room for learning
Schultz et al. (2012) [70]	Germany	provide empirical evidence for controlling the size and innovativeness of a hospital’s new health service and process portfolio	mixed-method	hospital	innovation (portfolio) management	innovation strategy
Schultz et al. (2017) [71]	Norway	to determine innovative readiness by looking at group climate	quantitative	hospital	group climate	innovative culture
Somech and Drach-Zahavy (2013) [72]	Israel	to study the relation between team composition of creative personalities, team creativity and climate for innovation	mixed-method	primary care	climate for innovation	innovative culture
Taylor et al. (2020) [73]	UK	explore the process through which staff innovates, when resources are scarce	qualitative	health care organizations	employee-driven innovation	innovative behavior
Thomas et al. (2005) [74]	UK	to identify what organizational features support innovation	qualitative	primary care	capacity to learn and innovate	room for learning
Timmermans et al. (2013) [75]	Belgium	to explore the influence of team learning on the implementation of innovations	quantitative	mental health institutions	team learning	room for learning
Urquhart et al. (2018) [76]	Canada	to examine the role of middle managers relevant to innovation implementation and how middle managers experience the implementation process	qualitative	hospital	middle manager rol in innovation	middle manager’s role
von Treuer et al. (2018) [77]	Australia	to examine the impact of organizational climate and leadership on organizational readiness for change	quantitative	nursing home	organizational climate leadership	innovative culture
Weatherford et al. (2018) [78]	USA	to identifying the characteristics of leaders of innovation	mixed-method	health care professionals	leaders of innovation competencies	innovative competencies
Zippel-Schultz and Schultz (2011) [79]	Germany	to analyze the role of business and project planning for innovation project success	quantitative	hospital	innovation program	innovation programm
Zuber and Moody (2018) [80]	USA	to explore how health centered design tools support innovation in health care	mixed method	health care professionals	innovation (supportive) environment	room for learning

### Definition of innovation readiness and frameworks

Six studies present a definition or description. Of these, only Benson [41] uses the term innovation readiness: “the degree to which an individual or organization is relatively earlier in adopting new ideas than other members of the system”. Where Benson emphasizes both the individual and organizational level, all five other studies present descriptions referring to the organizational level. Benson and Jaskyte et al. both emphasize the numerical aspect of innovation readiness: the timing of the adoption [41] and the number of adopted innovations [58]. Both Schultz et al. [71] and Von Treuer et al. [77] introduce the term readiness for change to describe “the likelihood of success in implementing an innovative change” [71]. Furthermore, Schultz et al. [70] as well as Joseph [60] mention the process aspect of innovation: the process of using knowledge [70] and innovation as a social process [60].

The authors of the other 38 studies use a variety of words addressing innovation readiness. The most frequently mentioned are: capacity for innovation [38, 39, 56, 78], innovation capacity [57], capacity to innovate [58, 62, 63], ability to innovate [40, 47, 79], organizational innovativeness [58], organization's innovation ability [48], innovation performance [52, 67], innovativeness of organizations [65], organizational innovation [80] and organization’s innovative potential [59].

Twenty-two of the 44 studies present their findings in a graphical framework consisting of the factors studied. Twenty-one studies present a partial framework displaying the specific studied factors that contribute to innovation readiness. For example, the framework of Birken et al. [42] demonstrates the relationship between top managers’ support and middle managers’ commitment to innovation implementation in health care organizations. Urquhart et al. [76] graphically display the understanding of middle managers’ roles in innovation implementation and the determinants of fulfilling these roles. Only Lombardi et al. [63] present a total organizational framework displaying a full set of factors that work together to promote innovation readiness of an entire organization. Their framework consists of 21 facilitating factors that intertwine to facilitate innovation in a health care organization with culture, structure and policy being the key organizational determinants.

### Factors contributing to innovation readiness

The authors MH and EV listed the factors of the 44 studies as described by the authors of the articles (see Table 1: Factor(s) studied (described by authors(s)). The list consisting of 44 factors contributing to innovation readiness were bundled at the level of the organization, the team and the individual. After discussion and reflection in the research team the decision was made to adjust and rename this bundling. The factors at the organizational level were preconditional and were named as main factor: strategic course for innovation. Main factor: leadership for innovation and climate for innovation clearly stood out as relevant elements on the team level. The individual level was named the main factor: commitment to innovation to reflect the content of the combined factors. The main category defines a main factor contributing to innovation readiness. The sub category gives detail to the main category. The process in the research team was an iterative reflective process and was either based on a suggestion by one author or on a group discussion with all authors.

**Fig. 2:**
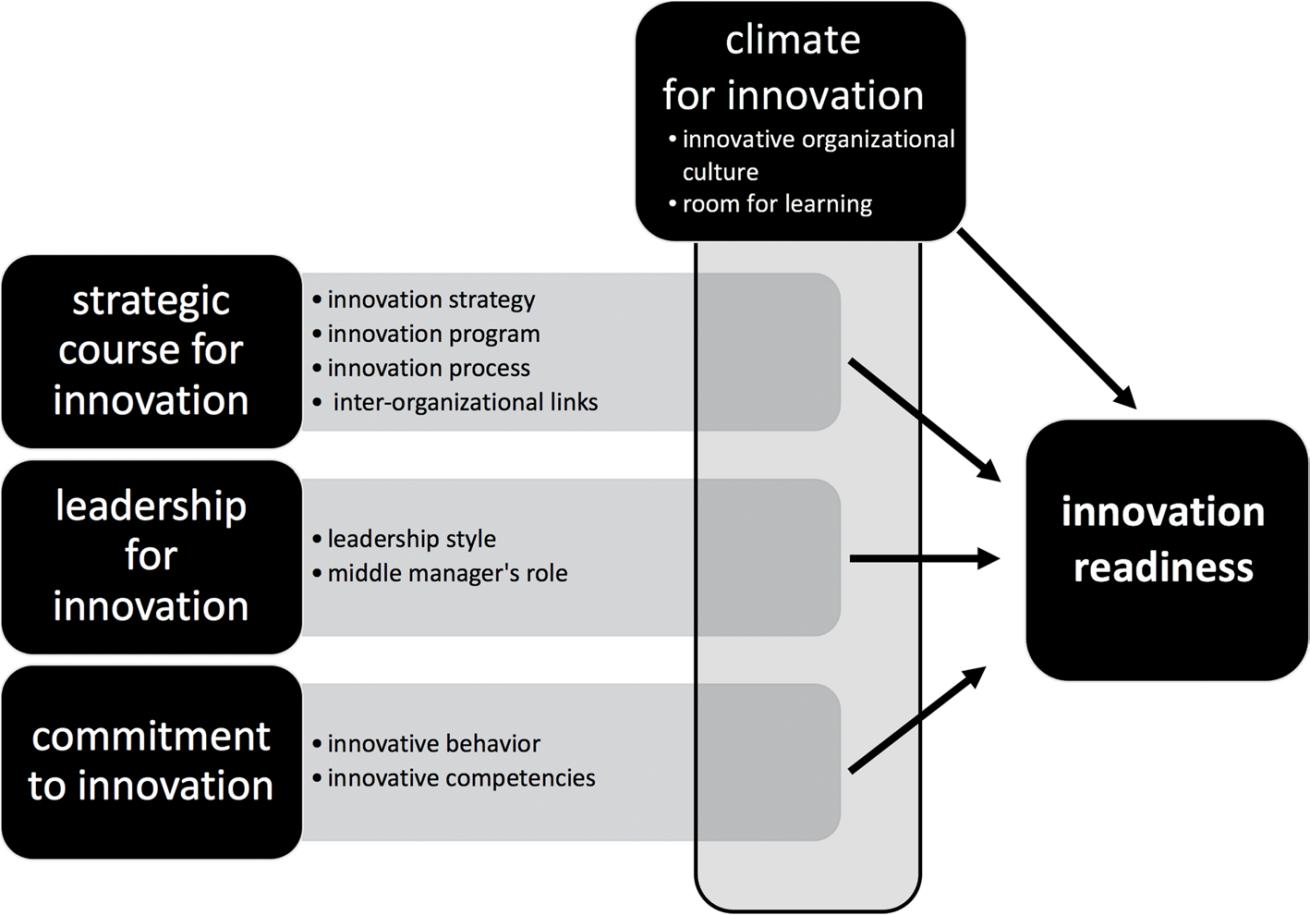
Factors contributing to innovation readiness

As a result the factors studied are categorized into four main factors: 1) strategic course for innovation, 2) climate for innovation, 3) leadership for innovation and 4) commitment to innovation. A general framework that represents the factors contributing to innovation readiness studied by the authors is presented in Fig. 2. The arrows in the framework depict the contribution of the four main factors to innovation readiness. The vertical presentation of the main factor climate for innovation illustrates its connectedness with the other three main factors. Each main factor consists of two or four sub-factors (see Table 2).

**Table 2:** (Main) factors contributing to innovation readiness researched in the included studies

Main factor	Sub-factor	Definition of factor
**Strategic course for innovation **		Strategic course for innovation refers to top management preparing the organization’s long-term direction to become innovation ready. It articulates the role and the importance of innovation for the organization in terms of strategic course and defines the allocation of resources between current operations and innovation
[50, 63, 70]	Innovation strategy	Innovation strategy concerns the alignment of innovation goals with the overall corporate strategy and prioritizes the desirable actions of employees
[39, 40, 52, 56, 57, 62, 66, 79]	Innovation program	Innovation program refers to the coordinated plans and actions that an organization undertakes to implement the innovation strategy
[39, 40, 57, 62, 66]	Innovation process	Innovation process concerns the policies and the steps that are taken in (part of) the organization from the idea of the innovation to sustaining the innovation
[43, 53]	Inter-organizational links	Inter-organizational links concern the relations of an organization with other organizations and how the organization uses these links to enhance innovation readiness
**Climate for innovation**		Climate for innovation concerns creating a supportive organizational environment that contributes to innovation readiness and focuses both at the team and organizational levels
[20, 46, 55, 58, 60, 61, 65, 71, 72, 77]	Innovative organizational culture	Innovative organizational culture concerns the way employees collectively think, behave and believe in relation to innovation readiness
[38, 44, 69, 74, 75, 80]	Room for learning	Room for learning concerns an environment that encourages the organization and employees to learn, reflect and acquire the knowledge and skills contributing to innovation readiness
**Leadership for innovation**		Leadership for innovation concerns the role of leadership of top and middle management to contribute to innovation readiness
[54, 64, 68]	Leadership style	Leadership style concerns the attitude and behavior of managers in leading the way to become innovation ready
[42, 45, 49, 51, 76]	Middle manager’s role	The middle manager’s role concerns the responsibilities and motivation of middle managers for innovation readiness
**Commitment to innovation**		Commitment to innovation concerns organizational actions aimed at the attitude, training and development of individual employees to support them in their individual readiness to get better at innovating
[48, 59, 67, 73]	Innovative behavior	Innovative behavior concerns employees undertaking innovative actions contributing to innovation readiness
[47, 78]	Innovative competencies	Innovative competencies concern the skills and motivation of employees to contribute to the innovation readiness of the organization

### Strategic course for innovation

Strategic course for innovation refers to top management preparing the organization’s long-term direction to become innovation ready. It articulates the role and the importance of innovation for the organization in terms of strategic course and defines the allocation of resources between current operations and innovation. The main factor consists of the sub-factors innovation strategy, innovation program, innovation process and inter-organizational links and is focused on the organizational level.

#### Innovation strategy

Innovation strategy concerns the alignment of innovation goals with the overall corporate strategy and prioritizes the desirable actions of employees and was included in three studies [50, 63, 70]. Two studies reported elements to design an effective innovation strategy: the characteristics of the sociopolitical context (e.g., legislation), the organization (e.g., the decision-making process), the adopting person (e.g., health professionals), the innovation (e.g., complexity) [50], organizational culture (e.g., values), organizational structure (e.g., processes, resources) and organizational policy (e.g., alignment of incentives) [63]. Additionally, Schultz et al. [70] studied what instruments an organization can use to harmonize employee goals with those of the organization and encourage employees to participate in the innovation strategy. The results demonstrated that formal instruments (e.g., rewards) and informal instruments (e.g., employee encouragement) influence the size and innovativeness of the innovation activities.

#### Innovation program

Innovation program refers to the coordinated plans and actions that an organization undertakes to implement the innovation strategy and is included in three studies [52, 56, 79]. All studies examined factors that are relevant for designing a successful innovation program. Glover et al. [52] examined the influence of hospital unit complexity on innovation. Hospital units are described as complex as they cannot be fully understood through linear thinking alone and interactions between team members can produce unpredictable behavior and generate new behavior. In order to achieve high levels of innovation in these innovation programs, units with higher levels of complexity should respond with lower staff autonomy (e.g., independence) and greater emphasis on performance orientation (e.g., demonstrating competence). Hunter et al. [56] studied a competition-based program designed to accelerate early-stage, innovative health care ideas from within the health care organization. Employees were encouraged to submit innovative ideas and if selected, they were provided with business, legal, technical and scientific project management support to help accelerate their projects. The program was modeled around four factors: small and dynamic project manager-led teams, low barriers to entry, emphasis on outreach and fostering innovators. The study found that project management and guidance for innovators were the most critical features of the innovation program. Business and project planning increase the success of innovation programs. Business planning ensures a fit between the innovation idea, the strategy and the competencies of the organization and project planning outlines the progress of a program [79].

#### Innovation process

Innovation process concerns the policies and the steps that are taken in (part of) the organization from the idea of the innovation to sustaining the innovation. Innovation process has been researched in five studies [39, 40, 57, 62, 66]. Three studies researched and identified factors relevant for the innovation process: the availability of quantitative evidence, inter-personal and inter-organizational networks, role of champions and senior management, the existence of a favorable inner and outer context [40, 66], organizational constraints [39] and knowledge management [62]. Atkinson et al. [39] examined two types of organizational constraints: heterarchical constraints (resistance from other professional groups and units in the organization) and hierarchical constraints (resistance from upper-level stakeholders and managers). The results demonstrate that teams address constraints at different stages of innovation by applying various tactics. Leal-Rodriguez et al. [62] studied the relationship between knowledge management and the effectiveness of the innovation process. According to them, knowledge management, defined as explicit and tacit components of organizational knowledge, leads to better innovation outcomes. Two studies researched the total innovation process. Hyrkas et al. [57] developed and tested a co-creation model for collaborative innovation, in which companies and health care professionals co-create future health care services. The results show that collaboration requires careful preparation, sector-specific knowledge and active efforts throughout the entire process. Reed et al. [66] studied the total process of innovation in health care organizations from strategic planning to implementation and found that the process of innovation is most dependent on organizational culture and leadership.

#### Inter-organizational links

Inter-organizational links concern the relations of an organization with other organizations and how the organization uses these links to enhance innovation readiness. Inter-organizational links are researched in two studies [43, 53]. Goes et al. [53] define inter-organizational links as “cooperative relationships among distinct but related organizations.” The relationships between innovation and four different but nonexclusive types of interorganizational links were studied: ‘structural links’ (organization is affiliated to corporate framework), ‘administrative links’ (e.g., contract management), ‘institutional links’ (link with institutional and trade associations) and ‘resource links’ (e.g., resource exchange). Results show that inter-organizational links provide an opportunity to exchange capabilities and knowledge between organizations and to enhance understanding of environmental trends. Bunn et al. [43] studied the researchers’ awareness of the organizational context in the collaboration of health care organizations and universities on innovating. The results show that to make scientific innovation research work in a health care setting, it is important for researchers to understand the experiences of staff, to engage them as active members of the research team and to support them to develop skills.

### Climate for innovation

Climate for innovation concerns creating a supportive organizational environment that contributes to innovation readiness and focuses both at the team and organizational levels. The main factor consists of the sub-factors innovative organizational culture and room for learning.

#### Innovative organizational culture

Innovative organizational culture concerns the way employees collectively think, behave and believe in relation to innovation readiness and is researched in ten studies [20, 46, 55, 58, 60, 61, 65, 71, 72, 77]. Innovative organizational culture is described as attitudes or behaviors of the team or the organization towards innovations [46, 55, 58]. Jacobs et al. [20] highlight the extent to which employees perceive that innovation is expected and rewarded by the organization, whereas Joseph [60] and Nieboer et al. [65] highlight innovative culture as “conditions” in which employees are supported in innovation.

A variety of factors that strengthen innovative culture were mentioned in the studies. Organizational support was frequently mentioned [20, 46, 55, 60, 61, 65] and is defined as time, training, innovationvalues fit (e.g., fit between the innovation and the values of innovation users) and implementation standards and policies. Investing in innovation standards and policies can be interpreted by employees as innovation being an organizational priority and contributing to a favorable perception of the culture of innovation [20, 61]. Furthermore, management support (e.g., coaching, priority setting) and leadership (e.g., guidance and preparing staff for change) are mentioned to enhance an innovative culture [46, 55, 60, 61, 65, 77]. An innovative culture is an environment that allows employees to inquire and question organizational practices [60], gives enough leeway to express creativity and allows employees to take risks, experiment and take advantage of opportunities [58]. Knowledge about how employees perceive the innovative culture can be used by management to determine if a group or organization is ready for innovation [71]. Somech et al. [72] detailed four dimensions of an innovative culture: vision, participative safety, task orientation and support for innovation. They studied team composition (combined individual creative personality and functional diversity), team creativity and climate for innovation and reported that team composition interacts with innovative culture.

#### Room for learning

Room for learning concerns an environment that encourages the organization and employees to learn, reflect and acquire the knowledge and skills contributing to innovation readiness and is researched in six studies as ‘capacity to learn and innovate’ [38, 44, 74] and ‘facilitation of learning’ [69, 75, 80]. Capacity to learn and innovate was researched in three studies [38, 44, 74]. The findings of Anvik et al. [38] demonstrate that everyday work practices of care professionals, besides planned learning situations, are an important point of departure for learning and innovation. Additionally, opportunities for people to reflect and learn at all levels of the organization as well as leadership that encourages employees to participate can increase the capacity to learn and innovate [74]. Learning communities (group learning in innovation clusters) provide opportunities to learn and can reduce the time required to move innovations into practice [44]. The facilitation of learning that enhances innovation readiness was researched in three studies [69, 75, 80]. Zuber et al. [80] observed that tools for creating innovative solutions, such as brainstorms and prototyping, increase the creative innovation potential of employees. The role of space in facilitating innovation is studied by Saidi et al. [69]. Attractive workspaces, different from the usual workplace, stimulate innovation through being “a space that enables interaction” because the productive interaction of different players inspires new ideas.

Furthermore, the influence of team learning on the implementation of innovation was studied. The results show that team learning will improve if the learning needs of a team are explored before the implementation of an innovation [75].

### Leadership for innovation

Leadership for innovation concerns the role of leadership of top and middle management to contribute to innovation readiness. The main factor consists of the sub-factors leadership style and middle manager’s role.

#### Leadership style

Leadership style concerns the attitude and behavior of managers in leading the way to become innovation ready. Leadership style is researched in three studies [54, 64, 68]. Transformational leadership, directed at inspirational motivation of employees by expressing a compelling vision [54], has a positive influence on the innovative behavior of employees [54, 64, 68], while transformational leadership encourages employees to think and solve problems innovatively [64]. In addition to transformational leadership, situational leadership (e.g., active participation from leaders) was considered important by staff as “leaders need to be present on the wards and know the skills of their employees” [68]. Gunzel-Jensen et al. [54] studied the interaction between different styles of leadership (transformational, transactional and empowering leadership) and reported that the combination of transformational and empowering leadership (aimed at increasing employees’ ability to make autonomous decisions when doing their job) has a strong positive influence on innovative behavior.

#### Middle manager’s role

The middle manager’s role concerns the responsibilities and motivation of middle managers for innovation readiness. The role is assessed in five studies [42, 45, 49, 51, 76]. Middle managers are important to make innovation implementation happen [49, 76], but their variety of roles and limited decision-making authority with respect to implementation hinders their innovative capacity [76]. García-Goñi et al. [51] studied the relation between motivation towards innovation and the involvement in the innovation process. As managers are more involved in the innovation process than other health professionals, they feel more motivated for the innovation than front-line employees. Chuang et al. [45] reported that middle manager support is encouraged by the fit between the innovation and the managers’ workplace priorities in combination with control over the implementation. Furthermore, Birken et al. [42] and Chuang et al. [45] reported that top managers can enhance middle manager commitment by expressing to middle managers that innovation implementation is an organizational priority and allocating policies and resources accordingly. In return, middle managers can utilize this support by asking for extra assistance in the execution of the innovation strategy [42].

### Commitment to innovation

Commitment to innovation concerns organizational actions aimed at the attitude, training and development of individual employees to support them in their individual readiness to get better at innovating. The main factor consists of the sub-factors innovative behavior and innovative competencies.

#### Innovative behavior

Innovative behavior concerns employees undertaking innovative actions contributing to innovation readiness and has been researched in four studies [48, 59, 67, 73]. Innovative behavior is stimulated by managerial support (e.g., motivation, rewards), cultural support (innovative climate) [48] and distributed leadership (leaders distribute leadership to employees and leave work decisions to employees) [59]. Furthermore, high-involvement HRM practices (e.g., training and development, performance feedback) positively influence innovative behavior as “employees perceive HRM practices as signals from the organization that innovative behaviors are appreciated” [67]. Organizational commitment and autonomy (feelings of control regarding one’s work) positively influence this relationship. Taylor et al. [73] studied the resourcing of innovation when employees innovate outside the context of innovative programs when resources are scarce. The results show that, where employees are driving innovation, they mobilize already present space, funding and staff at all levels of the organization and that the support of senior staff is crucial in facilitating access to resources.

#### Innovative competencies

Innovative competencies concern the skills and motivation of employees to contribute to the innovation readiness of the organization and has been researched in two studies [47, 78]. An increase in individual competences necessary for the use of information technology, typically a non-core competence of health care workers, resulted in a higher organizational ability to innovate [47]. Weatherford et al. [78] studied knowledge, skills and attitudes specific for leaders of innovation in health care to set up an education plan for innovation leaders. Five competency domains for leaders of innovation were identified: disruptive change (positive open attitude toward change), experimentation and design thinking, innovation and creativity, translating innovation into operations and risk taking.

## Discussion

Our study identified 10 sub-factors contributing to the innovation readiness of health care organizations that were clustered into four main factors: strategic course for innovation, climate for innovation, leadership for innovation and commitment to innovation (Fig. 2). Climate for innovation (*n* = 16) was the most studied, followed by strategic course for innovation (*n *= 13). Leadership for innovation (*n *= 8) and individual commitment to innovation (*n* = 6) were the least studied, despite the importance assigned in the literature to health care staff at all levels as key players in the development and implementation of innovations [73, 81, 82]. The factors found in this review show overlap with the literature about factors contributing to innovation readiness in business [83, 84] and health care [17]. The theme strategic course for innovation is reflected in elements such as innovation strategy [17, 83], project management [83] and planning [84]. Climate for innovation is reflected in elements such as the culture [17, 83] and context of the organization [84], whereas leadership for innovation is reflected in leadership [17] and commitment to innovation in support [84] and competencies [17]. Despite the overlap, there are also differences. Adams et al.’ s framework of the innovation management process [83], the ISO Innovation management system [84] and Nauta et al.’ s health care innovation management model [17] display portfolio management and internal and external communication as factors contributing to innovation readiness.

The majority (almost 90%; *n* = 36) of studies have been conducted since 2011, indicating that research into the innovation readiness of health care organizations is a rather new field. According to Weiner et al. [4], management consultants have written about organizational readiness for decades, although scientists have only recently focused attention on the importance of organizational readiness in supporting innovation in health settings. The author’s diverse personal and professional backgrounds reflect the fact that research into innovation readiness is conducted by various disciplines among which are health policy and management, innovation implementation, entrepreneurship, health care education, leadership, learning, medicine and art & design. This scoping review shows that the concept of innovation readiness in health care is described in the literature with a variety of terms and there does not seem to be a generally accepted definition of innovation readiness. Furthermore, it reveals that the likely interplay within and between the four main and sub-categories contributing to innovation readiness are understudied and understanding their interplay will support organizations in becoming better at innovating. Hardly any theoretical frameworks encompassing innovation readiness were used in the reviewed studies.

Almost half of the studies (*n* = 19) in this scoping review were performed in hospitals. The other half of the studies were undertaken in a variety of health care settings (e.g., nursing homes, mental health institutions, primary care and public health). Organizational characteristics (such as facility size), market characteristics (such as competition) [85, 86] and the educational level of staff [85] are found to influence innovative activities of organizations and might explain the larger representation of hospitals in this review. The question is whether research findings about innovation readiness can be transferred from one context to another. Besides the above-mentioned characteristics, the external setting and the roles played by external stakeholders (e.g., regulators) will impact the measures that organizations need to take to become innovation ready [87–91]. It might be worthwhile understanding how innovation readiness differs over the diverse contexts in health care.

Of the 44 studies reviewed, 21 researched factors contributing to innovation readiness in the implementation stage of the innovation process. The main stages of an entire innovation process are described as idea generation, idea selection, solution development, implementation, scale-up and diffusion [17]. Opportunities for innovation are sought, selected and developed in the first stages. In the implementation phase, the innovation is put into practice by health care professionals [92]. In the last stage, actions are taken to support full scale spread of the innovation in the organization. The focus in research on the implementation of innovations is reflected by the attention in research to heavily cited implementation models (Rogers [93] and Greenhalgh et al. [6]) and in international peerreviewed journals on the implementation of research. The emphasis on an evidence-based approach in care might have provoked an impulse for the implementation of innovation, although experience revealed that innovations developed elsewhere cannot simply be implemented in any other context [94]. Furthermore, the focus might be explained by the funding of national and regional governments to enhance the implementation and up-scaling of innovations to benefit specific target groups [10, 40, 95]. “A company’s capacity to innovate is only as good as the weakest link in its innovation value chain” [96]. Consequently, understanding innovation readiness in health care might benefit from research encompassing every stage of the innovation process.

This study has several strengths and limitations. In terms of strengths, it offers a unique contribution by presenting the state of the knowledge reflected in peer-reviewed literature from empirical studies, on factors contributing to innovation readiness in health care organizations. The study used a transparent and replicable review process. However, our study presents some limitations. First, it is subject to publication bias, since we only included studies published in peer-reviewed journals and excluded gray literature. Second, as there is no clear and consistent definition of innovation readiness, related search terms used for the search strategy can cause selection bias due to searching for specific words within the selected databases. Selection bias in the selection of studies was prevented by considering a broad range of words in relation to innovation readiness and reflection in the research team. Third, all but two studies (Pakistan and South Africa) were performed in the developed world and may impose a limitation on the findings of the study. The study increased the understanding of the determinants of innovation readiness and can support managers in health care organizations to decide on how to manage their efforts to become better at innovating.

## Conclusions

This scoping review has summarized four main factors contributing to innovation readiness in health care organizations: 1) strategic course for innovation 2) climate for innovation 3) leadership for innovation and 4) commitment to innovation. The factors contributing to innovation readiness (Fig. 2) seem to correspond to factors known in the literature. Research into the innovation readiness of health care organizations is a rather new field and lacks a generally accepted definition of innovation readiness, as well as a theoretical framework to guide research into innovation readiness. Future research could be directed towards defining the concept of innovation readiness and the development of a framework for innovation readiness to support managers in diverse health care settings to meet the challenges that health care organizations face. This review contributes to the first stage of theory building on factors contributing to innovation readiness. However, a better understanding of the interplay of factors contributing to innovation readiness in all stages of the innovation process and in diverse health care settings is needed.

## Supplementary Information

**Additional file 1.**

**Additional file 2.**

## Data Availability

The data that support the findings of this review are included in this published article. No primary data were collected.

## References

[1] Mulgan G, Albury D (2003). Innovation in the public sector. Strategy Unit, Cabinet Office.

[2] Williams I (2011). Organizational readiness for innovation in health care: some lessons from the recent literature. Health Serv Manag Res.

[3] Kievit PJ, Oomes J, Schoorl M, Bartels P (2018). The missing link: toward an assessment of innovation capacity in health care organizations. Int J Qual Innov.

[4] Weiner BJ, Clary AS, Klaman SL, Turner K, Alishahi-Tabriz A. Organizational Readiness for Change: What We Know, What We Think We Know, and What We Need to Know. In: Albers B, Shlonsky A, Mildon R, editors. Implementation Science 30. edn. Cham: Springer International Publishing; 2020. p. 101–144.

[5] Chaves BG, Briand C, Bouabida K. Innovation in healthcare organizations: Concepts and challenges to consider. Int J Health Res Innov. 2021a. 2021;9(1).

[6] Greenhalgh T, Robert G, Macfarlane F, Bate P, Kyriakidou O (2004). Diffusion of innovations in service organizations: systematic review and recommendations. Milbank Q.

[7] Smith M, Busi M, Ball P, Van Der Meer R. Factors influencing an organisation’s ability to manage innovation: A structured literature review and conceptual model. Managing Innovation: What Do We Know About Innovation Success Factors? 2019;69-90.

[8] du Plessis MJ, Pretorius MW. Does my organization need an innovation management function?: Towards a model for evaluation. In: 2018 Portland International Conference on Management of Engineering and Technology (PICMET): 2018. IEEE; 2018. p. 1-10.

[9] Harsanto B. Innovation in hospital: Learning from Malcolm Baldrige Awards latest winners in healthcare. In: International Conference Health, Science And Technology (ICOHETECH). 2021;2021:62-64.

[10] Nolte E, Organization WH. How do we ensure that innovation in health service delivery and organization is implemented, sustained and spread? 2018.

[11] Begun JW, Jiang HJ (2020). Health care management during Covid-19: Insights from complexity science. NEJM Catalyst Innovations in Care Delivery.

[12] Tiffany C, Oomer M, Tavarez V. How Innovative Community Responses to COVID-19 Support Healthy Aging. FSG; 2020. p. 40.

[13] Lyng HB, Ree E, Wibe T, Wiig S (2021). Healthcare leaders’ use of innovative solutions to ensure resilience in healthcare during the Covid-19 pandemic: a qualitative study in Norwegian nursing homes and home care services. BMC Health Serv Res.

[14] Janssen M, Stoopendaal AMV, Putters K (2015). Situated novelty: Introducing a process perspective on the study of innovation. Res Policy.

[15] Kelly CJ, Young AJ (2017). Promoting innovation in healthcare. Future healthcare journal.

[16] Arora A, Wright A, Cheng M, Khwaja Z, Seah M (2021). Innovation Pathways in the NHS: An Introductory Review. Ther Innov Regul Sci.

[17] Nauta F, Crombach M. Innovatiescan Nederlandse zorg: Een inventarisatie van innovatiemanagement in de Nederlandse zorgsector. 2011.

[18] Labitzke G, Svoboda S, Schultz C (2014). The Role of Dedicated Innovation Functions for Innovation Process Control and Performance - An Empirical Study among Hospitals. Creat Innov Manag.

[19] Verleye K, Gemmel P (2013). Innovation in the elderly care sector – at the edge of chaos. Journal of Management & Marketing in Healthcare.

[20] Jacobs SR, Weiner BJ, Reeve BB, Hofmann DA, Christian M, Weinberger M. Determining the predictors of innovation implementation in healthcare: a quantitative analysis of implementation effectiveness. BMC Health Services Res. 2015;15(1):1-13. 10.1186/s12913-014-0657-3 PMC4307151 25608564

[21] Peterson C, Hamilton C, Hasvold P. From innovation to implementation – eHealth in the WHO European Region; 2016.

[22] Colldén C, Hellström A (2018). Value-based healthcare translated: a complementary view of implementation. BMC Health Serv Res.

[23] Guarcello C, de Vargas ER (2020). Service Innovation in Healthcare: A Systematic Literature Review. Lat Am Bus Rev.

[24] Thijssen SV, Jacobs MJ, Swart RR, Heising L, Ou CX, Roumen C. The barriers and facilitators of radical innovation implementation in secondary healthcare: a systematic review. J Health Organ Manag. 2021. 10.1108/JHOM-12-2020-0493 PMC10430798 34910413

[25] Weintraub P, McKee M (2019). Leadership for innovation in healthcare: an exploration. Int J Health Policy Manag.

[26] Zuber C, Weberg D (2020). Frameworks for Leading Frontline Innovation in Health Care: Failure, Microclimates, and Leadership. Nurse Lead.

[27] Marjanovic S, Altenhofer M, Hocking L, Chataway J, Ling T (2020). Innovating for improved healthcare: Sociotechnical and innovation systems perspectives and lessons from the NHS. Science and Public Policy.

[28] Leedham-Green K, Knight A, Reedy GB (2021). Success and limiting factors in health service innovation: a theory-generating mixed methods evaluation of UK projects. BMJ Open.

[29] Hyrkas P, Lunkka N, Reponen J, Suhonen M (2022). Factors enabling innovation activities: combining perspectives of personnel and management of a public university hospital. INTERNATIONAL JOURNAL OF INNOVATION AND LEARNING.

[30] Snyder-Halpern R (2001). Indicators of organizational readiness for clinical information technology/systems innovation: a Delphi study. Int J Med Inform.

[31] Zerfass A (2005). Innovation readiness. Innovation Journalism.

[32] Robert G, Greenhalgh T, MacFarlane F, Peacock R. Organisational factors influencing technology adoption and assimilation in the NHS: a systematic literature review. Report for the National Institute for Health Research Service Delivery and Organisation programme 2009.

[33] Arksey H, O'Malley L (2005). Scoping studies: towards a methodological framework. Int J Soc Res Methodol.

[34] Peters MDJ, Marnie C, Tricco AC, Pollock D, Munn Z, Alexander L, McInerney P, Godfrey CM, Khalil H (2020). Updated methodological guidance for the conduct of scoping reviews. JBI Evidence Synthesis.

[35] Tricco AC, Lillie E, Zarin W, O'Brien KK, Colquhoun H, Levac D, Moher D, Peters MDJ, Horsley T, Weeks L (2018). PRISMA Extension for Scoping Reviews (PRISMA-ScR): Checklist and Explanation. Ann Intern Med.

[36] Peters MDJ, Godfrey C, McInerney P, Khalil H, Larsen P, Marnie C, Pollock D, Tricco AC, Munn Z (2022). Best practice guidance and reporting items for the development of scoping review protocols. JBI Evidence Synthesis.

[37] Miles MB, Huberman AM, Saldaña J. Qualitative data analysis: A methods sourcebook. Sage publications; 2018.

[38] Anvik C, Vedeler JS, Wegener C, Slettebo A, Odegard A (2020). Practice-based learning and innovation in nursing homes. J Work Learn.

[39] Atkinson MK, Singer SJ. Managing Organizational Constraints in Innovation Teams: A Qualitative Study Across Four Health Systems. Med Care Res Rev. 2021;78(5):521-36. 10.1177/1077558720925993 PMC8483614 32552540

[40] Barnett J, Vasileiou K, Djemil F, Brooks L, Young T. Understanding innovators' experiences of barriers and facilitators in implementation and diffusion of healthcare service innovations: a qualitative study. BMC Health Services Res. 2011;11(1):1-12. 10.1186/1472-6963-11-342 PMC3265424 22176739

[41] Benson T. Digital innovation evaluation: user perceptions of innovation readiness, digital confidence, innovation adoption, user experience and behaviour change. BMJ Health Care Inform. 2019;26(1):1-6. 10.1136/bmjhci-2019-000018 PMC7062319 31039121

[42] Birken SA, Lee SY, Weiner BJ, Chin MH, Chiu M, Schaefer CT (2015). From strategy to action: how top managers' support increases middle managers' commitment to innovation implementation in health care organizations. Health Care Manage Rev.

[43] Bunn F, Goodman C, Corazzini K, Sharpe R, Handley M, Lynch J, Meyer J, Dening T, Gordon AL (2020). Setting Priorities to Inform Assessment of Care Homes' Readiness to Participate in Healthcare Innovation: A Systematic Mapping Review and Consensus Process. Int J Environ Res Public Health.

[44] Carpenter D, Hassell S, Mardon R, Fair S, Johnson M, Siegel S, Nix M (2018). Using Learning Communities to Support Adoption of Health Care Innovations. Jt Comm J Qual Patient Saf.

[45] Chuang E, Jason K, Morgan JC (2011). Implementing complex innovations: factors influencing middle manager support. Health Care Manage Rev.

[46] Cramm JM, Strating MM, Bal R, Nieboer AP. A large-scale longitudinal study indicating the importance of perceived effectiveness, organizational and management support for innovative culture. Soc Sci Med. 2013;83:119-24. 10.1016/j.socscimed.2013.01.017 23415046

[47] Dohan MS, Green M, Tan J (2017). The impact of healthcare informatics competencies on dynamic capabilities: A multilevel study of paramedic services. Health Policy Technol.

[48] Emiralioglu R, Sonmez B (2021). The relationship of nursing work environment and innovation support with nurses' innovative behaviours and outputs. J Nurs Manag.

[49] Engle RL, Lopez ER, Gormley KE, Chan JA, Charns MP, Lukas CV (2017). What roles do middle managers play in implementation of innovative practices?. Health Care Manage Rev.

[50] Fleuren M, Wiefferink K, Paulussen T (2004). Determinants of innovation within health care organizations: literature review and Delphi study. Int J Qual Health Care.

[51] García-Goñi M, Maroto A, Rubalcaba L (2007). Innovation and motivation in public health professionals. Health Policy.

[52] Glover WJ, Nissinboim N, Naveh E (2020). Examining innovation in hospital units: a complex adaptive systems approach. BMC Health Serv Res.

[53] Goes JB, Park SH (1997). Interorganizational links and innovation: The case of hospital services. Acad Manag J.

[54] Gunzel-Jensen F, Hansen JR, Jakobsen MLF, Wulff J (2018). A Two-Pronged Approach? Combined Leadership Styles and Innovative Behavior. Int J Public Adm.

[55] Helfrich CD, Weiner BJ, McKinney MM, Minasian L (2007). Determinants of implementation effectiveness: adapting a framework for complex innovations. Med Care Res Rev.

[56] Hunter RB, Winston FK, Dehel P, Oh K, Nicklas J, Hartung H (2021). SPRINTing to Innovation: Children's Hospital of Philadelphia's Strategic Approach to Discovering Its Untapped Innovation Potential. Acad Med.

[57] Hyrkas P, Haukipuro L, Vainamo S, Iivari M, Sachinopoulou A, Majava J (2020). Collaborative innovation in healthcare: a case study of hospitals as innovation platforms. International Journal of Value Chain Management.

[58] Jaskyte K, Dressler WW (2005). Organizational culture and innovation in nonprofit human service organizations. Adm Soc Work.

[59] Jonsson TF, Unterrainer CM, Kahler HG. Do autonomous and trusting hospital employees generate, promote and implement more ideas? The role of distributed leadership agency. Eur J Innov Manag 18.

[60] Joseph ML (2015). Organizational culture and climate for promoting innovativeness. J Nurs Adm.

[61] Keown OP, Parston G, Patel H, Rennie F, Saoud F, Al Kuwari H, Darzi A (2014). Lessons from eight countries on diffusing innovation in health care. Health Aff (Millwood).

[62] Leal-Rodriguez AL, Roldan JL, Leal AG, Ortega-Gutierrez J (2013). Knowledge management, relational learning, and the effectiveness of innovation outcomes. Serv Ind J.

[63] Lombardi MM, Spratling RG, Pan W, Shapiro SE (2018). Measuring Organizational Capacity to Accelerate Health Care Innovation in Academic Health Centers. Qual Manag Health Care.

[64] Masood M, Afsar B (2017). Transformational leadership and innovative work behavior among nursing staff. Nurs Inq.

[65] Nieboer AP, Strating MM (2012). Innovative culture in long-term care settings: the influence of organizational characteristics. Health Care Manage Rev.

[66] Reed P, Conrad DA, Hernandez SE, Watts C, Marcus-Smith M. Innovation in patient-centered care: lessons from a qualitative study of innovative health care organizations in Washington State. BMC Fam Pract. 2012;13(1):1-9. 10.1186/1471-2296-13-120 PMC3538717 23241305

[67] Renkema M, De Leede J, Van Zyl LE (2021). High-Involvement HRM and Innovative Behaviour: The Mediating Roles of Nursing Staff's Autonomy and Affective Commitment. J Nurs Manag.

[68] Rokstad AM, Vatne S, Engedal K, Selbaek G (2015). The role of leadership in the implementation of person-centred care using Dementia Care Mapping: a study in three nursing homes. J Nurs Manag.

[69] Saidi T, de Villiers K, Douglas TS (2017). The sociology of space as a catalyst for innovation in the health sector. Soc Sci Med..

[70] Schultz C, Zippel-Schultz B, Salomo S (2012). Hospital innovation portfolios: key determinants of size and innovativeness. Health Care Manage Review.

[71] Schultz JS, Sjøvold E, Andre B. Can group climate explain innovative readiness for change? Journal of Organizational Change Management. 2017;30(3):440-52.

[72] Somech A, Drach-Zahavy A (2013). Translating Team Creativity to Innovation Implementation: The Role of Team Composition and Climate for Innovation. J Manag.

[73] Taylor R, Fuller A, Halford S, Lyle K, Teglborg AC (2020). Translating employee-driven innovation in healthcare: Bricolage and the mobilization of scarce resources. Public Money & Management.

[74] Thomas P, McDonnell J, McCulloch J, While A, Bosanquet N, Ferlie E (2005). Increasing capacity for innovation in bureaucratic primary care organizations: a whole system participatory action research project. Ann Fam Med.

[75] Timmermans O, Van Linge R, Van Petegem P, Van Rompaey B, Denekens J (2013). A contingency perspective on team learning and innovation in nursing. J Adv Nurs.

[76] Urquhart R, Kendell C, Folkes A, Reiman T, Grunfeld E, Porter GA (2018). Making It Happen: Middle Managers' Roles in Innovation Implementation in Health Care. Worldviews Evid Based Nurs.

[77] von Treuer K, Karantzas G, McCabe M, Mellor D, Konis A, Davison TE, O'Connor D (2018). Organizational factors associated with readiness for change in residential aged care settings. BMC Health Serv Res.

[78] Weatherford B, Bower KA, Vitello-Cicciu J (2018). The CNO and Leading Innovation: Competencies for the Future. Nurs Adm Q.

[79] Zippel-Schultz B, Schultz C (2011). Mediated and Moderated Effects of Business and Project Planning on Innovation Projects in Hospitals. Creativity and Innovation Management.

[80] Zuber CD, Moody L (2018). Creativity and Innovation in Health Care: Tapping Into Organizational Enablers Through Human-Centered Design. Nurs Adm Q.

[81] Busch-Casler J, Haubner S, Pinkwart A (2021). Employee involvement in innovation activities in hospitals: How perception matters. Health Serv Manag Res.

[82] PPalumbo R. Engaging to innovate: an investigation into the implications of engagement at work on innovative behaviors in healthcare organizations. J Health Organ Manag. 2021. 10.1108/JHOM-02-2021-0072 34170095

[83] Adams R, Bessant J, Phelps R (2006). Innovation management measurement: A review. Int J Manag Rev.

[84] ISO 56002:2019 Innovation management system - Guidance: ISO International Organization for Standardization; 2019.

[85] Castle NG (2001). Innovation in nursing homes: which facilities are the early adopters?. Gerontologist.

[86] Felix H, Dayama N, Morris ME, Pradhan R, Bradway C (2021). Organizational Characteristics and the Adoption of Electronic Health Records Among Nursing Homes in One Southern State. J Appl Gerontol.

[87] Najmaei A (2014). Towards an Integrative Model for Management of Organization's Total Innovation: Insights from the Strategic-Process View Towards an Integrative Model for Management of Organization's Total Innovation: Insights from the Strategic-Process View. The IUP Journal of Knowledge Management..

[88] Fu Q, Saleh MA (2021). Review of Innovation Management Frameworks and Assessment Tools. International Journal of Mechanical and Industrial Engineering.

[89] Flessa S, Huebner C (2021). Innovations in Health Care-A Conceptual Framework. Int J Environ Res Public Health.

[90] Verkerk MJ, VitaValley, Owls F. Iedereen beter: een toolbox voor zorginnovatie. Houten: Bohn Stafleu van Loghum; 2019.

[91] Tidd J (2021). A review and critical assessment of the ISO56002 innovation management systems standard: Evidence and limitations. Int J Innov Manag.

[92] Fleuren MA, Paulussen TG, Van Dommelen P, Van Buuren S (2014). Towards a measurement instrument for determinants of innovations. Int J Qual Health Care.

[93] Rogers EM, Singhal A, Quinlan MM. Diffusion of innovations. In: An integrated approach to communication theory and research. edn. Routledge; 2014. p. 432-48.

[94] Council for Public Health and Society. No evidence without context. About the illusion of evidence‐based practice in healthcare. 2017.

[95] Parston G, McQueen J, Patel H, Keown OP, Fontana G, Al Kuwari H, Darzi A (2015). The Science And Art Of Delivery: Accelerating The Diffusion Of Health Care Innovation. Health Aff (Millwood).

[96] Hansen MT, Birkinshaw J (2007). The Innovation Value Chain. Harv Bus Rev.

