# Supplementary material for: Factors contributing to innovation readiness in health care organizations: a scoping review
Source: BMC Health Serv Res. 2022 Aug 5;22:997. doi: 10.1186/s12913-022-08185-x (PMC9354428; doi:10.1186/s12913-022-08185-x)
Supplement: Supplementary file 2 — Additional file 2. [file 12913_2022_8185_MOESM2_ESM.docx]

**Additional file 2 Detailed search terms used in the PubMed search***

| **Step** | **Search terms** |
| --- | --- |
| **1** | ***Concept term: innovation readiness*** |
|  | "change read*"[Text Word] OR "change capabilit*"[Text Word] OR "change capacit*"[Text Word] OR "dynamic capa*"[Text Word] OR "innovation capacit*"[Text Word] OR "innovation capabilit*"[Text Word] OR "Innovation management"[Text Word] OR “innovation maturity"[Text Word] OR "innovation performance*"[Text Word] OR "Innovation potential"[Text Word] OR "Innovation process*"[Text Word] OR "innovation read*"[Text Word] OR “innovation transformation*"[Text Word] OR "organizational capacit*"[Text Word] OR "organisational capacit*"[Text Word] OR "organizational capabilit*"[Text Word] OR "organisational capabilit*"[Text Word] OR "Organizational innovativeness"[Text Word] OR "Organisational innovativeness"[Text Word] OR "Organizational potential"[Text Word] OR "Organisational potential"[Text Word] OR "organizational read*"[Text Word] OR "organisational read*"[Text Word] OR "System readiness for innovation*"[Text Word] OR "readiness for innovation"[Text Word] OR "diffusing innovation*"[Text Word] OR "Innovation implementation"[Text Word] OR "innovation diffusion*"[Text Word] OR "implementation of innovation*"[Text Word] OR "innovation adoption*"[Text Word] OR "adopt innovation*"[Text Word] OR "health care innovation*"[Text Word] OR "healthcare innovation*"[Text Word] OR "hospital innovation*"[Text Word] OR "organizational innovation*"[Title/Abstract] OR "organisational innovation*"[Title/Abstract] OR "Diffusion of Innovation*"[Title/Abstract] |

* Detailed search strategies used in the other databases (Cinahl and Web of Science) are available upon request
